# Low oxygen levels contribute to improve photohydrogen production in mixotrophic non-stressed Chlamydomonas cultures

**DOI:** 10.1186/s13068-015-0341-9

**Published:** 2015-09-17

**Authors:** Jose Luis Jurado-Oller, Alexandra Dubini, Aurora Galván, Emilio Fernández, David González-Ballester

**Affiliations:** Departamento de Bioquímica y Biología Molecular, Facultad de Ciencias, Universidad de Córdoba, Campus de Rabanales, Edif. Severo Ochoa, 14071 Córdoba, Spain; Biosciences Center, National Renewable Energy Laboratory (NREL), 15013 Denver West Parkway, Golden, CO 80401 USA

**Keywords:** Acetate, Algae, Biofuels, Biomass, Chlamydomonas, DCMU, Hydrogen, Low light, Oxygen

## Abstract

**Background:**

Currently, hydrogen fuel is derived mainly from fossil fuels, but there is an increasing interest in clean and sustainable technologies for hydrogen production. In this context, the ability of some photosynthetic microorganisms, particularly cyanobacteria and microalgae, to produce hydrogen is a promising alternative for renewable, clean-energy production. Among a diverse array of photosynthetic microorganisms able to produce hydrogen, the green algae *Chlamydomonas reinhardtii* is the model organism widely used to study hydrogen production. Despite the well-known fact that acetate-containing medium enhances hydrogen production in this algae, little is known about the precise role of acetate during this process.

**Results:**

We have examined several physiological aspects related to acetate assimilation in the context of hydrogen production metabolism. Measurements of oxygen and CO_2_ levels, acetate uptake, and cell growth were performed under different light conditions, and oxygenic regimes. We show that oxygen and light intensity levels control acetate assimilation and modulate hydrogen production. We also demonstrate that the determination of the contribution of the PSII-dependent hydrogen production pathway in mixotrophic cultures, using the photosynthetic inhibitor DCMU, can lead to dissimilar results when used under various oxygenic regimes. The level of inhibition of DCMU in hydrogen production under low light seems to be linked to the acetate uptake rates. Moreover, we highlight the importance of releasing the hydrogen partial pressure to avoid an inherent inhibitory factor on the hydrogen production.

**Conclusion:**

Low levels of oxygen allow for low acetate uptake rates, and paradoxically, lead to efficient and sustained production of hydrogen. Our data suggest that acetate plays an important role in the hydrogen production process, during non-stressed conditions, other than establishing anaerobiosis, and independent of starch accumulation. Potential metabolic pathways involved in hydrogen production in mixotrophic cultures are discussed. Mixotrophic nutrient-replete cultures under low light are shown to be an alternative for the simultaneous production of hydrogen and biomass.

**Electronic supplementary material:**

The online version of this article (doi:10.1186/s13068-015-0341-9) contains supplementary material, which is available to authorized users.

## Background

Chlamydomonas hydrogenase HYDA1 is able to catalyze hydrogen (H_2_) production under anaerobic conditions using protons (H^+^) and electrons as substrates; oxygen (O_2_) is a strong inhibitor of both activity and expression [1, 2]. HYDA1 is localized in the chloroplast and its physiological electron donor is the plastidic ferredoxin, PETF or FDX1 (FDX1 throughout) [3–5]. Three pathways for H_2_ production can be described in Chlamydomonas depending on the electron source and the electron transport pathway to HYDA1. With two of these pathways, the electrons come from photosynthetic electron transport, and they are termed photosystem II (PSII)-dependent and PSII-independent pathways. In the first case, the electrons come from water photolysis at the level of PSII, and O_2_ is generated as a by-product [6–8]. In the PSII-independent pathway, the electrons come from NADPH [9, 10], and reach the plastoquinone (PQ) pool via the NAD(P)H:plastoquinone oxidoreductase, NDA2 [11–14]. Degradation of starch is proposed as the main source for NADPH [15, 16]. Both electrons routes connect to FDX1 prior to final electron donation to HYDA1. The third pathway mobilizes electrons from the fermentative degradation of endogenous compounds, such as pyruvate derived from starch [16–19]. Fermentative H_2_ production is linked to the activity of the pyruvate:ferredoxin oxidoreductase (PFR), which can donate electrons to HYDA1 via FDX1 [20].

Chlamydomonas is capable of growing photoautotrophically using light and CO_2_ as the sole carbon source, or mixotrophically (acetate and light), using acetate as a carbon source. The observation that in some algal species adapted to light and anaerobiosis, H_2_ photoproduction is enhanced by the presence of acetate in the media was discovered several decades ago [17, 21–24]. These former studies established that under illumination algae can consume the acetate available in the medium and subsequently release H_2_ and CO_2_. In the dark, however, acetate has no effect on H_2_ production, and acetate uptake is not detected. Despite these initial studies, little has been done recently regarding the role of acetate in the production of H_2_ by algae in nutrient-replete media [25, 26].

In this work, we have examined the physiology of photohydrogen production in acetate-containing cultures, and we discuss the importance of controlling acetate assimilation through a fine tune of the O_2_ and light intensity levels for efficient and sustainable production of H_2_.

## Results

### Acetate-containing media and low light are non-stressful conditions that elicits H_2_ production

**Fig. 1:**
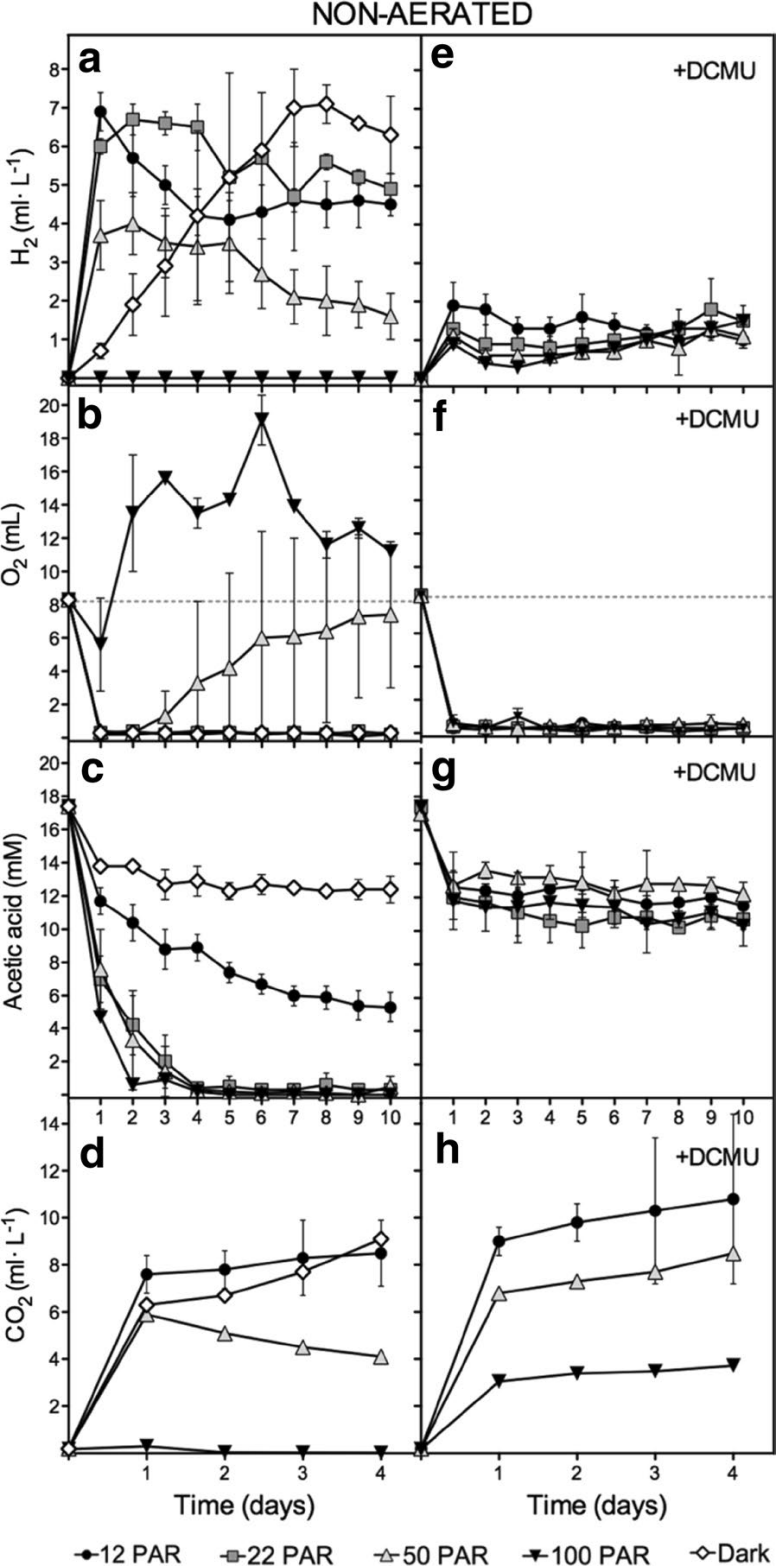
Determination of H_2_, O_2_, acetic acid, and CO_2_ levels in non-aerated cultures incubated at different light intensities. H_2_ (**a**, **e**), O_2_ (**b**, **f**) and CO_2_ (**d**, **h**) levels were determined for the headspaces. Acetic acid (**c**, **g**) was determined in the media. Panels **a** to **d** correspond to measurements done in TAP media, whereas panels **e** to **h** correspond to measurements done in TAP media supplemented with DCMU. Represented data are average from at least three independent experiments

Hermetically sealed vessels containing 100 ml of low cell density (10 µg chl./ml; ~3 million cells/ml) Chlamydomonas cells cultured in Tris–Acetate–Phosphate (TAP) were placed under four different light conditions (12, 22, 50 and 100 µmol photons m^−2^ s^−1^; hereafter, 12 PAR, 22 PAR, 50 PAR, and 100 PAR) and dark. In this work, we will term 12 and 22 PAR as Low Light (LL), 50 PAR as Moderate Light (ML), and 100 PAR as High Light (HL). The headspaces of the culture vessels (40 ml) were not purged, and therefore atmospheric air was present in all vessels at the beginning of the experiments. H_2_, CO_2_ and O_2_ accumulation in the headspace, chlorophyll content, and acetate consumption were measured daily during 10-day experiments (Fig. 1 and Additional file 1: Fig. S1).

Cultures incubated under LL and ML produced H_2_ after 24 h, at rates of 6.9, 6.0, and 3.7 ml L^−1^ day^−1^ for 12, 22, and 50 PAR, respectively (Fig. 1a). After 48 h, little or no H_2_ production was observed under these light intensities. Interestingly, the H_2_ accumulation observed under light did not reach a steady-state level, but rather a small decrease was observed after the maximal H_2_ accumulation was reached. The H_2_ decrease was most evident under the 12 PAR condition. This observation is likely due to H_2_ uptake activity. Cultures under 100 PAR resulted in no H_2_ production. Finally, dark cultures had lower initial H_2_-production rates relative to 12–50 PAR conditions (average less than 1 ml L^−1^ day^−1^), although production was sustained for more than 7 days. Final H_2_ accumulation in dark cultures, after 8 days, was similar to that obtained under LL after 24–48 h. The atmospheric levels of O_2_ initially present in the headspaces were totally consumed after 24 h for all conditions except 100 PAR (Fig. 1b). O_2_ levels in the headspace remained close to zero for 12, 22 PAR and the dark conditions over the 10 days of the experiment. However, under the 50 PAR conditions, O_2_ accumulation was observed in the headspace after 3 days. At 100 PAR, O_2_ evolved above the atmospheric level, indicating that PSII activity overtook respiration rates. Acetate uptake also showed light dependence under our experimental conditions (Fig. 1c). Under 22–100 PAR, all the acetic acid contained in the media was essentially consumed after 2–4 days. However, we observed slower acetate uptake kinetics under 12 PAR and dark conditions. After 10 days, there was still acetic acid in the media (5.3 mM and 9.5 mM for 12 PAR and dark, respectively). CO_2_ quickly accumulated after 24 h in all tested conditions except 100 PAR (Fig. 1d). The media pH remained stable over the 10 days (7.5–7.8). Finally, the chlorophyll concentration increased significantly under 22–100 PAR, whereas there was very little or no increase at 12 PAR and in the dark, respectively (Additional file 1: Fig. S1A).

These data indicate that acetate-containing cultures grown under ≤50 PAR and moderately low cell density can rapidly consume O_2_ from the headspaces (for a 100:40 medium: headspace v/v ratio), reaching anaerobiosis and producing H_2_ after 24 h. Maximal H_2_ production rates were inversely correlated with light intensities. However, dark cultures did not experience a rapid H_2_-production phase but rather a slow, continued, and sustained level of H_2_ production. This indicates that light is crucial to induce the rapid H_2_ production kinetics observed under LL and ML.

### Aerations of cultures can double H_2_ photoproduction

**Fig. 2:**
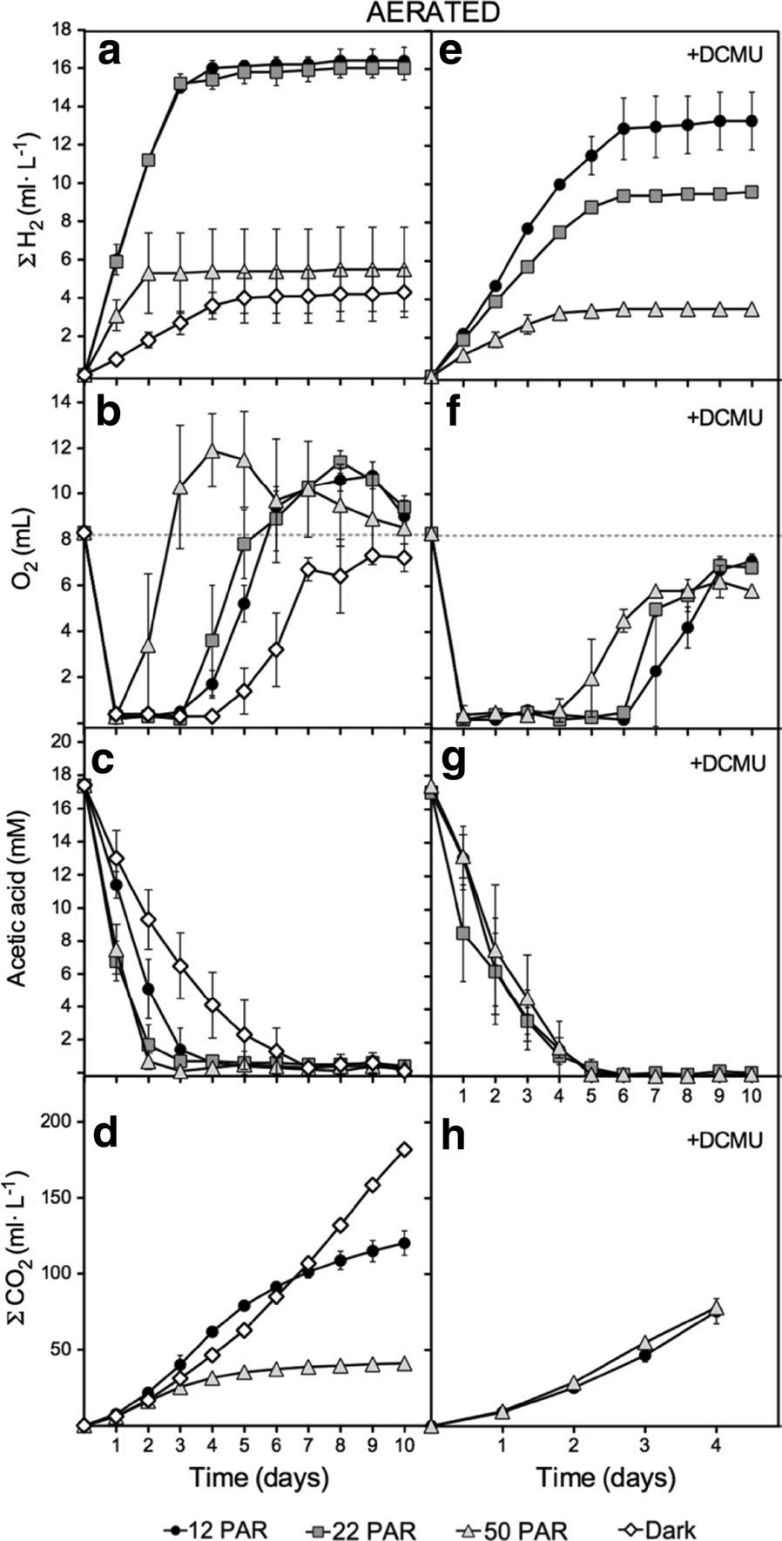
Determination of H_2_, O_2_, acetic acid, and CO_2_ levels in aerated cultures at different light intensities. H_2_ (**a**, **e**), O_2_ (**b**, **f**) and CO_2_ (**d**, **h**) levels were determined for the headspaces. Acetic acid (**c**, **g**) was determined in the media. Panels **a** to **d** correspond to measurements done in TAP media, whereas panels **e** to **h** correspond to measurements done in TAP media supplemented with DCMU. Aeration was performed by simply opening the caps of the vessels for 5 min in an sterile atmosphere every 24 h. H_2_, O_2_, and CO_2_ accumulation in the headspace were measured daily before aeration. For H_2_ and CO_2_, the accumulative productions were plotted. O_2_ levels are plotted as daily measurements. Represented data are average from at least three independent experiments

The same experimental conditions described above were also used to monitoring H_2_ production in aerated vessels. Aeration was performed by opening the caps of the vessels for 5 min, in a sterile atmosphere, every 24 h. H_2_, CO_2_ and O_2_ accumulation in the headspace, acetate consumption, and chlorophyll content were measured daily before aeration during 10-day experiments (Fig. 2; Additional file 1: Fig. S1). H_2_ and CO_2_ levels are plotted as total production, whereas O_2_ levels are plotted as daily measurements. Chlorophyll increased steadily with the intensity of light (Additional file 1: Fig. S1), whereas no changes were observed for pH, which was around 7.6–7.8.

The H_2_ production in aerated cultures under LL was 2.4 times higher than in non-aerated cultures under the same light conditions (Figs. 2a vs 1a). For cultures under 50 PAR and dark, the aeration either slightly increased or reduced H_2_ production, respectively, relative to non-aerated cultures. Under LL, unlike the non-aerated vessels, H_2_ production was sustained for 3 days. Afterwards, very little H_2_ production was observed. For LL grown cultures, atmospheric levels of O_2_ entering in the vessels daily were completely consumed during the first 3 days (Fig. 2b). By the 4th day, increased levels of O_2_ were detected in the headspace indicating that the O_2_ consumption rates were progressively decreasing. Similar results were observed for cultures under 50 PAR, although appearance of O_2_ in the headspaces was detected earlier as the light intensity was increased. For all light intensities, except darkness, headspace O_2_ accumulation exceeded the initial O_2_ atmospheric levels, indicating net O_2_ photoproduction (Fig. 2b). Unlike the non-aerated cultures, all the acetate initially contained in the media was eventually consumed in the aerated cultures under all the conditions tested (Fig. 2c). More specifically, in the light, acetic acid was consumed after 2–3 days. We observed a strong correlation among the H_2_ production, the level of O_2_ in the headspace, and the consumption of acetate, indicating that these three processes are closely interconnected. H_2_ was produced as long as acetate was present in the media. Once acetate was consumed, O_2_ was accumulated and H_2_ production ceased. The higher the light intensity, the faster the acetate was consumed, the faster O_2_ accumulated in the headspace, and the faster H_2_ production ceased. Notably, increasing the light intensity not only reduced the length of the H_2_-production phase but also reduced the daily H_2_-production rates (e.g., 5 ml L^−1^ day^−1^ for 12PAR vs 2.6 ml L^−1^ day^−1^ for 50 PAR). However, in the dark, H_2_ production dropped drastically reinforcing the idea that light is crucial for the H_2_ production rates observed in the light. CO_2_ production was significantly higher for all the conditions tested when comparing to non-aerated cultures (Figs. 2d vs 1d).

### Release of H_2_ partial pressure highly promotes H_2_ production in mixotrophic LL cultures

**Fig. 3:**
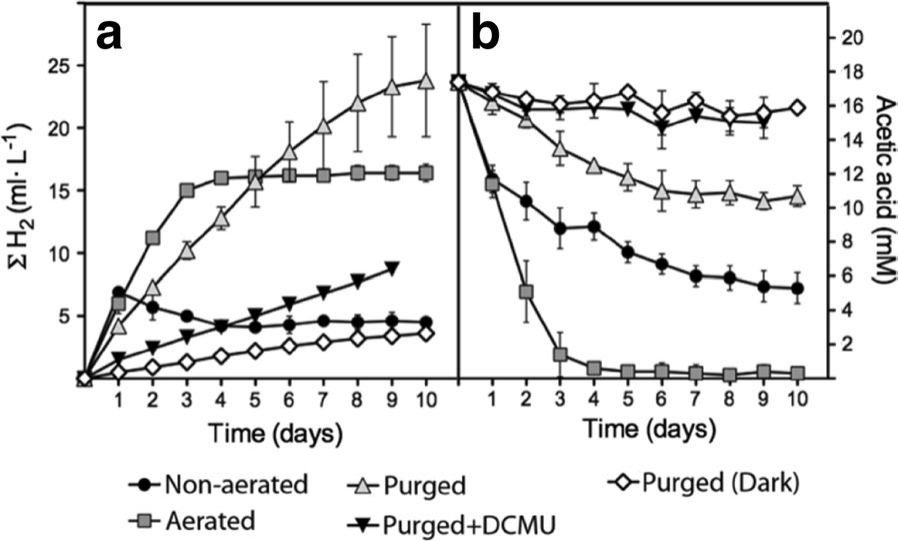
Determination of H_2_ (**a**) and acetic acid (**b**) on daily N_2_-purged cultures under 12 PAR and in the dark. Cultures incubated under 12 PAR, 12 PAR + DCMU and in the dark were purged daily for 5 min with N_2_ gas. For comparison purposes, aerated and non-aerated 12 PAR culture conditions are also included in the graphic. Other details are as in Fig. 2. Represented data are average from at least three independent experiments

Two possibilities might explain the higher H_2_ accumulation observed in aerated cultures relative to non-aerated cultures: (1) the increased acetic acid uptake observed in aerated cultures may result in higher H_2_ production, or (2) release of the H_2_ partial pressure from the headspace may favor the equilibrium displacement of the reversible reaction catalyzed by hydrogenases toward H_2_ production and preventing H_2_ uptake [27]. When the headspaces of 12 PAR grown cultures were N_2_-purged daily, H_2_-production rates during the first 3 days were similar to those observed in aerated cultures (Fig. 3a). However, N_2_-purged cultures kept producing H_2_ for 10 days, while aerated cultures stopped producing H_2_ by the 4th day (Fig. 3b). Total H_2_ production over the 10 days in the purged cultures was 1.4 times higher than in aerated cultures. Interestingly, acetate uptake in purged cultures was severely impaired compared to aerated cultures (Fig. 3b). Purged cultures consumed only around 7 mM of acetic acid but produced more H_2_ than aerated cultures, which consumed all the acetate initially present in the media (17.4 mM). These data indicate that enhanced H_2_ production under aeration is mainly due to partial H_2_ pressure release and not to the increased acetate uptake rates.

To further test which of the two initial hypotheses were true, we added to non-aerated cultures pure O_2_ daily to the headspaces at atmospheric levels using a syringe (Additional file 2: Fig. S2). Cultures remained hermetically sealed for 9 days without releasing the H_2_ partial pressure. The results showed high acetate uptake rates, but this did not improve H_2_ production, which was similar to that obtained for non-aerated cultures. O_2_ from the headspaces was consumed for 4 days; after 5 days, O_2_ started to accumulate. This indicates that despite the fact that cultures reached anoxia for 4 days and that acetate was totally consumed, the H_2_ partial pressure in the headspaces blocked further H_2_ production.

### Acetic acid and O_2_ supplementation leads to sustained H_2_ production under LL conditions

**Fig. 4:**
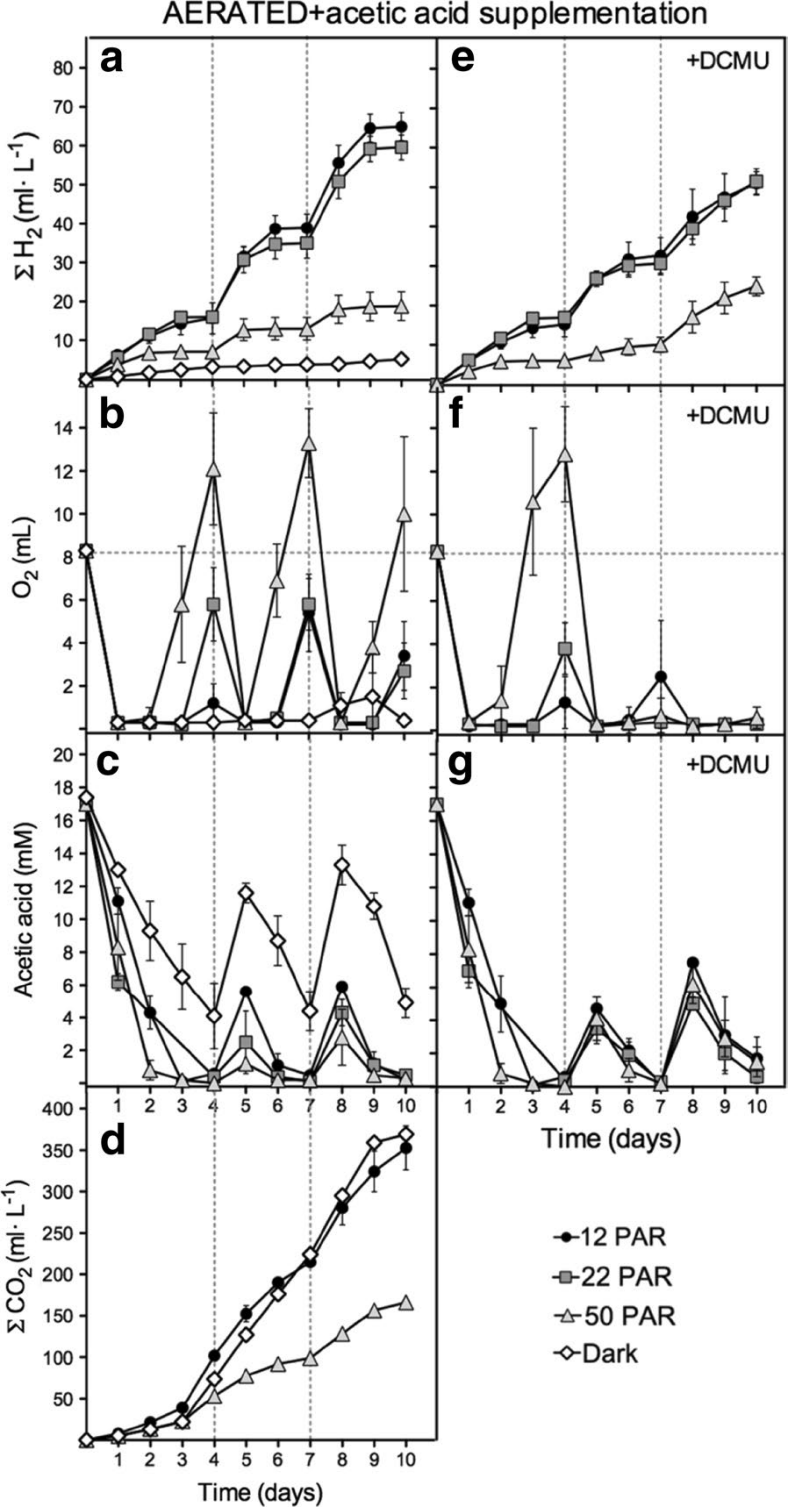
Determination of H_2_, O_2_, CO_2_, and acetic acid in aerated cultures supplemented with acetic acid at different light intensities. H_2_ (**a**, **e**), O_2_ (**b**, **f**) and CO_2_ (**d**) levels were determined for the headspaces. Acetic acid (**c**, **g**) was determined in the media. Panels **a** to **d** correspond to measurements done in TAP media supplemented with acetic acid (8.5 mM), whereas panels **e** to **f** correspond to measurements done in TAP media supplemented simultaneously with acetic acid (8.5 mM) and DCMU. Acetic acid and DCMU supplementations were done on days 4 and 7 (indicated with a *gray dashed line*). Other details are as in Fig. 2. Represented data are average from at least three independent experiments

Cultures were subjected to identical experimental conditions described for aerated cultures for 4 days. After 4 days, cultures consumed most of the initial acetate (17.4 mM) contained in the media and did not significantly produce H_2_ afterwards (Fig. 2a). The addition of extra acetic acid (8.7 mM final concentration) on the 4th day promoted a second boost of H_2_ production under all light conditions except on cultures kept in the dark (Fig. 4a). After acetic acid supplementation, H_2_ production was sustained for another one (50 PAR) or two (12 and 22 PAR) days before ceasing again. Addition of a second dose of acetic acid on the 7th day (once H_2_ production stopped and acetate was consumed again) promoted a 3rd H_2_-production boost. Supplementation with acetic acid did not significantly change the pH of the media over the 10 days of the experiment, which remains about 7.5–7.8. In agreement with our previous data, LL grown cultures produced more H_2_ than 50PAR cultures. At 12 PAR, the maximal H_2_ production rate was 21.2 ml per day, and net total accumulation was 14.9 and 4.0 times higher compared to non-aerated and aerated without supplementation cultures, respectively. Similar to aerated cultures, we observed a good correlation between H_2_ production, acetate uptake, and O_2_ consumption in the headspace (Fig. 4a–c). Addition of extra acetic acid to the medium increased CO_2_ production under all conditions tested, compared to non-supplemented cultures (Figs. 4d vs 2d). Supplementation with acetic acid without performing aeration resulted in cell death (data not shown). This is likely due to the low acetic acid uptake observed in the absence of O_2_ (see below section), which in turn results in acidification of the media (pHs of the dead cultures were of 5.6). Overall, the data showed that H_2_ production can be sustained in fed-batch type bioreactors under LL with both aeration and acetic acid supplementation.

### Acetate uptake depends on O_2_ availability. The inhibitory effect of DCMU on H_2_ production under LL is linked to O_2_ availability and impairment of acetate uptake

To determine the role of PSII activity in the observed H_2_ production under our experimental conditions, DCMU was added to the cultures.

The addition of DCMU to the non-aerated cultures incubated under 12–50 PAR produced a substantial reduction of H_2_ accumulation compared to the cultures containing no DCMU (78.8–69.5 % reduction at 24 h) (Fig. 1e vs a). We also observed an impairment in acetate uptake in non-aerated cultures treated with DCMU under all light intensities; the acetate concentration initially dropped but no significant acetate uptake was observed after 24–48 h (Fig. 1g). Interestingly, in aerated cultures, this reduction in H_2_ production when using DCMU (Fig. 2e) was significantly smaller than the one obtained for non-aerated cultures (e.g., 19.2 and 40.1 % at 12 and 22 PAR, respectively). Moreover, addition of DCMU to aerated cultures caused a much lower inhibition of the acetate uptake than in non-aerated cultures with DCMU (Figs. 2g vs 1g). Indeed, acetate was totally consumed in aerated cultures with DCMU after 4–5 days, while in non-aerated cultures, about 10 mM of acetate remained in the media after 10 days. When daily purged cultures under 12 PAR were supplemented with DCMU, both acetate uptake and H_2_ production dropped severely (Fig. 3). DCMU caused a 64 % inhibition of H_2_ production, and acetic acid uptake was practically inexistent. The effects of DCMU on H_2_ production and acetate uptake in purged cultures resemble the observed in non-aerated cultures. Finally, simultaneous addition of both acetic acid and DCMU on the 4th and 7th days in aerated cultures resulted in partial inhibition of H_2_ production (e.g., 21.5 % reduction for 12 PAR) relative to cultures supplemented with acetic acid but without DCMU (Fig. 4e vs a). However, acetate uptake was not significantly affected in these cultures (Fig. 4g vs c). These data are very similar to those obtained with aerated cultures without extra acetic acid supplementation.

Two main conclusions can be obtained from these data. First, we show that acetate uptake depends on the availability of O_2_. In the presence of DCMU, daily aeration of the cultures can supply the O_2_ needed to consume all the acetate from the media (Fig. 2g), whereas in non-aerated or purged vessels with DCMU, the lack of O_2_ inhibits acetate uptake (Fig. 1g). The initial drop in the acetate levels observed in non-aerated cultures containing DCMU after 24–48 h is likely due to the initial presence of O_2_ in the non-purged headspace; as O_2_ level decreased, no more acetate was consumed (Fig. 1g). Moreover, acetate uptake does not depend on the PSII activity *per se*. However, indirectly, the PSII activity contributes providing O_2_. This can be observed in non-aerated and aerated cultures without DCMU (Figs. 1c, 2c): daily aeration of the cultures can supply the O_2_ needed to consume all the acetate from the media, whereas in non-aerated vessels the PSII activity is the only source of O_2_ that enables acetate uptake. The light dependence of acetate uptake observed in both non-aerated and aerated cultures without DCMU can be explained by the relative activity of the PSII and the availability of the photo-evolved O_2_. Previous observations describing acetate consumption in the light and termed as photoassimilation of acetate (or acetate photometabolism) [21, 28] must be linked, at least partially, to the photogeneration of O_2_ but not to the ATP availability.

Second, we demonstrate that DCMU can affect differently H_2_ production in LL cultures. The addition of DCMU to non-aerated and purged cultures grown under 12 PAR caused very similar effects on inhibition of H_2_ production (72.4 vs 64 %, respectively) (Figs. 1e, 3a) and on acetate uptake (essentially blocked) (Figs. 1g, 3b). The effect of DCMU, however, is clearly different in aerated cultures cultivated under 12 PAR (with or without supplementation with acetic and DCMU) where H_2_ production was only partially inhibited (nearly 20 %) (Figs. 2e, 4e) and acetate uptake was not severely impaired (Figs. 2g, 4g). Note that the degree of inhibition of the H_2_ production caused by DCMU correlates with the O_2_ availability and the rates of acetate uptake. The higher H_2_ inhibition was obtained when O_2_ availability and acetate uptake were very low.

Finally, addition of DCMU to non-aerated cultures grown under HL elicited a small H_2_ production (Fig. 1e). H_2_ production was only possible under HL when DCMU was present in the medium. The presence of this inhibitor was crucial to reach anaerobiosis under HL (Fig. 1f), unlike in the 12–50 PAR grown cultures. Similar results have been found in autotrophic sulfur-depleted cultures [29].

### Mobilization of starch reserves are not linked to H_2_ production in acetate-containing media under LL

Starch mobilization has been proposed to contribute to H_2_ production in mixotrophic nutrient-replete cultures via the PSII-independent pathway [16, 17, 21]. It has been suggested that under aerobic conditions acetate would first stimulate starch accumulation, which would be later degraded under anoxic conditions and provide the PQ pool with reductive equivalents. Hence, either the starch reserves present prior the beginning of the experiments or those accumulated during the experiments might potentially contribute to H_2_ production under LL.

**Fig. 5:**
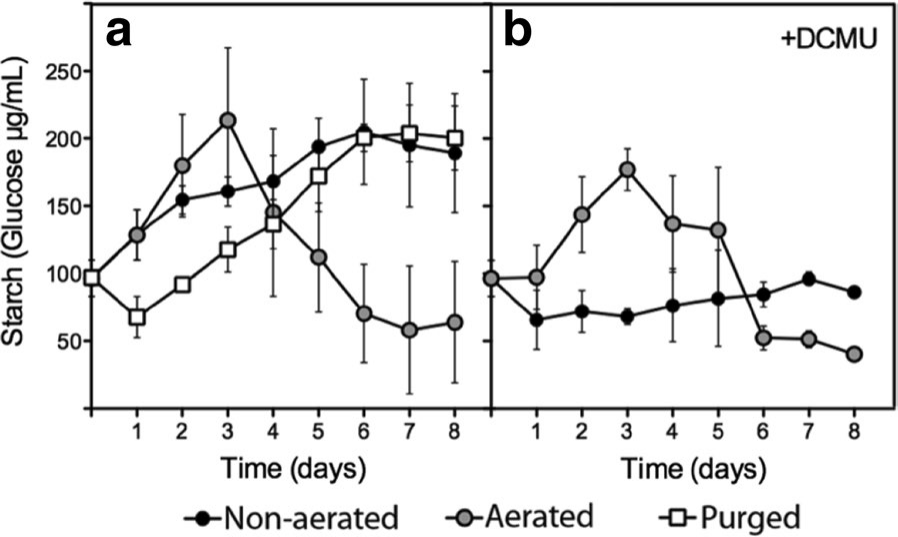
Starch evolution in aerated and non-aerated cultures under 12 PAR. Starch measurements were performed daily in cultures without DCMU (**a**) and with DCMU (**b**). Represented data are average from at least three independent experiments

We analyzed the starch accumulation patterns in the LL cultures (Fig. 5). In non-aerated cultures, starch accumulated progressively doubling its initial concentration at the end of the 8-day experiment (Fig. 5a). Similarly, in daily purged cultures under LL, starch initially decreased after 24 h but then accumulated during 6 days reaching a steady level afterwards (Fig. 5a). In aerated cultures, an initial starch accumulation phase was observed followed by a degradation phase. We observed a good synchronization between the disappearance of acetate from the media (Fig. 2c) and the starch degradation phase in aerated cultures under LL (Fig. 5a). The addition of DCMU to non-aerated cultures clearly impaired starch accumulation (Fig. 5b), likely reflecting the poor acetate uptake measured in these cultures (Fig. 1g). The addition of DCMU to aerated cultures had only a minor effect on the starch accumulation pattern (Fig. 5b), which is in agreement with the poor inhibition of acetate uptake observed in these cultures (Fig. 2g), and demonstrate that starch accumulation does not require PSII activity but acetate uptake.

Overall, we observed no correlation between starch accumulation/degradation patterns and H_2_ production because the starch accumulation phases correlated with the maximal H_2_-production rates in all cases, and there was no further H_2_ production when starch was degraded (e.g., aerated cultures, see Figs. 5 vs 2 and 2e).

### Comparing H_2_ production in LL mixotrophic nutrient-replete and S-depleted cultures

H_2_ production under LL mixotrophic S-depleted conditions was tested using the previously described experimental conditions.

Non-aerated, S-depleted cultures at 12PAR (without purged head space) showed an initial H_2_-production phase resembling that obtained with S-replete cultures. After 7 days, a second H_2_-production boost was observed (Supplemental Fig. 3a). While the first H_2_-production boost might share similar physiological processes with nutrient-replete cultures, the second H_2_ boost could be more specific to S-deprivation physiology. Interestingly, non-aerated S-depleted cultures presented higher tolerance to the inhibitory effect of the H_2_ partial pressure than nutrient-replete cultures. Maximal H_2_ accumulation in non-aerated S-depleted cultures (13.7 ml L^−1^) was 2 times higher than in non-aerated S-replete cultures (6.9 ml L^−1^), but similar to the production obtained in aerated S-replete cultures (16.4 ml L^−1^) (Supplemental Fig. 3b). S-depleted aerated cultures produce no H_2_ (Additional file 3: Fig. S3B) because cells were unable to consume the O_2_ that daily entered in the headspaces and anaerobiosis never occurred (data not shown) revealing that O_2_ consumption rates in S-depleted cultures are lower than in S-replete cultures. S-depleted daily purged cultures showed H_2_ production rates quite similar to those of S-replete purged cultures (Additional file 3: Fig. S3C).

Overall, the results showed that although S starvation can improve H_2_ production in non-aerated cultures, similar levels of H_2_ can be obtained in S-replete, aerated cultures in a shorter period of time. Moreover, S-depleted, purged cultures did not show any significant advantage over S-replete purged cultures. Hence, under our experimental conditions, removal of S from the cultures does not contribute a significant advantage over nutrient-replete media under LL.

## Discussion

### H_2_ partial pressure critically inhibits H_2_ production

Hydrogenases have a reversible nature and are capable of both biosynthesizing H_2_ and dissociating it into H^+^ and electrons. This latter activity is commonly known as H_2_ uptake. As any chemical reaction, the equilibrium between the biosynthesis and uptake of H_2_ depends on the concentrations of substrates and products. This would imply that H_2_ accumulation within bioreactors increases H_2_ partial pressure in the headspace, while reducing H_2_ biosynthetic rates to eventually cease any net hydrogenase bioproduction activity.

Inhibition of the Chlamydomonas H_2_ production by injection of different amounts of pure H_2_ in the headspaces has previously been observed by others [21, 27]. Moreover, Mignolet et al. [14] demonstrated how H_2_ elimination from hermetic cultures by flushing with N_2_ can enhance H_2_ production. Finally, Kosourov et al. [27] also studied the effect of different volumes of purged headspaces on H_2_ production in Chlamydomonas cultures under S- and phosphorous-deprivation. Here, we certainly confirm that the release of the H_2_ accumulated in the headspace greatly enhances H_2_ production, even when this release is accompanied by O_2_ supplementation to the cultures (Fig. 2a). Daily release of the H_2_ accumulated in the headspaces of the bioreactors vessels, through either aeration, or purging with N_2_, resulted in H_2_ production 2.4 and 3.4 times higher than in non-aerated cultures, respectively (Fig. 3a). In our experimental designs, release of the H_2_ partial pressure was executed every 24 h. Since non-aerated cultures did not evolve substantial H_2_ after 24 h (Fig. 1a), we conclude that under our nutrient-replete LL conditions, the equilibrium between H_2_ production and H_2_ uptake was reached before the 24-h aeration events. In fact, H_2_ uptake can be observed after 24 h in LL, non-aerated cultures (Fig. 1a). For dark non-aerated cultures, the equilibrium is reached after 7–8 days (Fig. 1a). However, for both non-aerated LL and dark cultures, the maximal accumulation of H_2_ in the headspaces reached about 1.9 % (0.76 ml H_2_ out of 40 ml headspace). Hence, we assume that 1.9 % is near the maximal percentage of H_2_ that can be accumulated under our specific experimental nutrient-replete conditions.

Interestingly, in S-depleted, non-aerated cultures (Additional file 3: Fig. S3A), the maximal H_2_ accumulation was higher than in non-aerated S-replete cultures and reached 3.2 % of the headspace. This could indicate that different physiological conditions can probably alter the substrates availability of the hydrogenases (H^+^ and electrons) exerting different equilibrium pressures towards H_2_ formation.

Overcoming the inhibition of H_2_ accumulation within bioreactors must be dealt with to optimize H_2_ production. Utilization of a larger gas to liquid phase ratios or ventilation systems that liberate H_2_ partial pressure are straightforward ways to overcome the hydrogenase reversibility. However, this may lead the production of highly diluted H_2_, which may in turn reduce the potential for commercial applications. Utilization of bioreactors covered with materials, such as metallacarboranes, with high reversible capacity for binding H_2_ at low temperatures [30] might be a way to avoid H_2_ uptake.

### Production of both biomass and H_2_ is possible in non-stressed Chlamydomonas cultures

Hydrogenase sensitivity to O_2_ is probably the main drawback for sustained H_2_ photoproduction in photosynthetic organisms. Reduction of net O_2_ levels can be accomplished by reducing PSII activity and maintaining high rates of mitochondrial respiration. At physiological level, nutrient stresses (in the presence of acetate) are widely employed strategies to establish this condition in Chlamydomonas. Sulfur limitation is the most widely employed [6], although phosphorous [31], nitrogen [32] and magnesium [33] deficiencies have been used successfully to attain H_2_ production in Chlamydomonas. These approaches imply a two-stage process: first biomass is grown under aerobic conditions in nutrient-replete media, and then a H_2_ production phase can be induced by removing nutrients and O_2_ from the media. This procedure normally requires a solid–liquid separation step of biomass and culture medium after the growth phase, followed by several washing steps and O_2_ purging processes [6, 34, 35]. Continuous or semi-continuous regimes of cultivation [36–38] or re-addition of nutrients to the medium [39] are alternatives to the solid–liquid separation steps, which extend the sustainability of H_2_ production. However, all these protocols normally require high-energy inputs, do not support algae biomass and H_2_ production simultaneously, are time-consuming and reduce culture viability.

In this work, we show an alternative straightforward methodology for H_2_ production in nutrient-replete media under LL. This methodology neither requires initial high cell density cultures nor the removal of nutrients from the media. Moreover, under LL and in the presence of acetate, the atmospheric O_2_ is totally depleted after 24 h, which makes the purging process, required in other strategies, unnecessary. Finally, H_2_ production can start within 24 h avoiding the typical lag phase (2–8 days) observed under nutrient-depleted cultures [15, 29, 31–33]. Overall, this strategy opens up new possibilities for production of both H_2_ and biomass, the latter being important for further downstream biotechnological applications.

Here, we demonstrated that Chlamydomonas, nutrient-replete, mixotrophic, cultures grown under LL (<50 PAR) can reach anoxia after 24 h and produce H_2_, highlighting the inverse relationship between H_2_ production rates and light intensities. Similar results have been previously reported by Degrenne et al. [26] who studied the induction of anoxia and H_2_ production in mixotrophic Chlamydomonas cultures using batch and continuous mode bioreactors under different light regimes. Here, we have extended these previous studies using cultures under different light intensities and oxygenic regimes. We have described in detail how light intensity, O_2_ availability, acetate uptake, and H_2_ production are processes closely linked to each other. Here, we have shown that O_2_ allows acetate uptake and cell growth. Providing O_2_ to batch mode cultures under LL allowed us to achieve simultaneously H_2_ production and biomass (Fig. 2a; Additional file 1: Fig. S1). Moreover, in aerated fed-batch mode bioreactors, non-continuous supplementation with acetic acid and O_2_ greatly enhanced H_2_ production (Fig. 4a). Likely continuous mode bioreactors, with continuous supply of acetic acid and low levels of O_2_ (plus other basic nutrients), would allow for sustained H_2_ and biomass production.

In our study, maximal H_2_ production was obtained when cultures under 12PAR were not aerated but N_2_-purged daily. The purging procedure allowed prolonged H_2_ production (10 days in purged cultures vs 3 days in aerated cultures) (Fig. 3) with a substantial reduction of the acetate consumption, which is of interest for potential large-scale productions. However, N_2_-purged cultures did not grow and biomass did not increase, likely because O_2_ levels were too low. Optimization of these procedures must be achieved to obtain both maximal H_2_ and biomass production. For example, purging gases that include low concentration of O_2_ can be finely tuned to allow for high H_2_ production rates and cell growth.

### Roles of acetate and oxygen in the production of H_2_ under LL in nutrient-replete cultures

Most of the studies related to H_2_ production in Chlamydomonas have been performed with S-depleted acetate-containing media [6, 10, 34, 40]. The goal of these studies, however, did not focus on identifying the role of acetate during H_2_ production. When autotrophic Chlamydomonas cultures are used to produce H_2_ in either nutrient-replete [41] or S-depleted media [29, 35, 42], H_2_ production is low compared with media containing acetate, which highlight the importance of acetate in the context of H_2_ production under these conditions. From studies using nutrient-replete and S-depleted media, it is widely assumed that acetate facilitates H_2_ production by helping to establish anoxia in the cultures through its oxidation via the Tricarboxylic Acid Cycle (TCA) and oxidative phosphorylation [6, 17, 21, 26, 34, 43]. The addition of acetate to Chlamydomonas cultures decreases photosynthesis efficiency, net O_2_ evolution, and CO_2_ fixation, as well as promotes the transition from state I to state II and mitochondrial respiration [44–47]. All these factors help to establish anoxia in sealed cultures. Repression of CO_2_ fixation may also contribute to H_2_ production by reducing the competition for electrons, for HYDA1, at the level of FDX1 [10]. In any case, the role of acetate in stimulation of H_2_ production in the light has not yet been carefully examined.

We have shown that acetate uptake rates are not directly proportional to H_2_ production rates (e.g., purged cultures vs aerated cultures, Fig. 3a, b). Indeed, the faster the acetate is consumed, the faster O_2_ accumulates (once acetate is depleted from the media) and the faster H_2_-production phase is inhibited (e.g., Fig. 2a–c). On the other hand, we have shown that acetate uptake is highly dependent on O_2_ availability (e.g., comparing aerated and non-aerated cultures with DCMU, Figs. 1g, 2g). Under severe anoxic conditions, such as in non-aerated cultures with DCMU or daily purged cultures with DCMU, there is no acetate uptake and H_2_ production is very limited (Figs. 1g, e, 3a, b). This suggests that a minimal threshold of acetate uptake is needed to support H_2_ production and that the role of acetate must rely on something else than just the establishment of anoxic condition. Moreover, paradoxically, O_2_ is a key substrate that under limiting concentrations allows acetate uptake and thereby H_2_ production. Acetate uptake does not directly depend on the PSII activity *per se* (Fig. 2g); however, PSII activity is crucial to provide O_2_ to hermetically sealed cultures and allow acetate uptake (e.g., Fig. 1c). Overall, our results suggest that an important aspect to be considered when producing H_2_ in mixotrophic Chlamydomonas cultures is that optimal H_2_ production does not rely on reaching very high respiration rates or establishing severe anoxic conditions but rather on sustaining relatively low acetate uptake rates under hypoxic conditions. LL is an optimal physiological condition where O_2_ production is high enough to allow low acetate uptake rates but low enough to prevent hydrogenase inhibition.

**Fig. 6:**
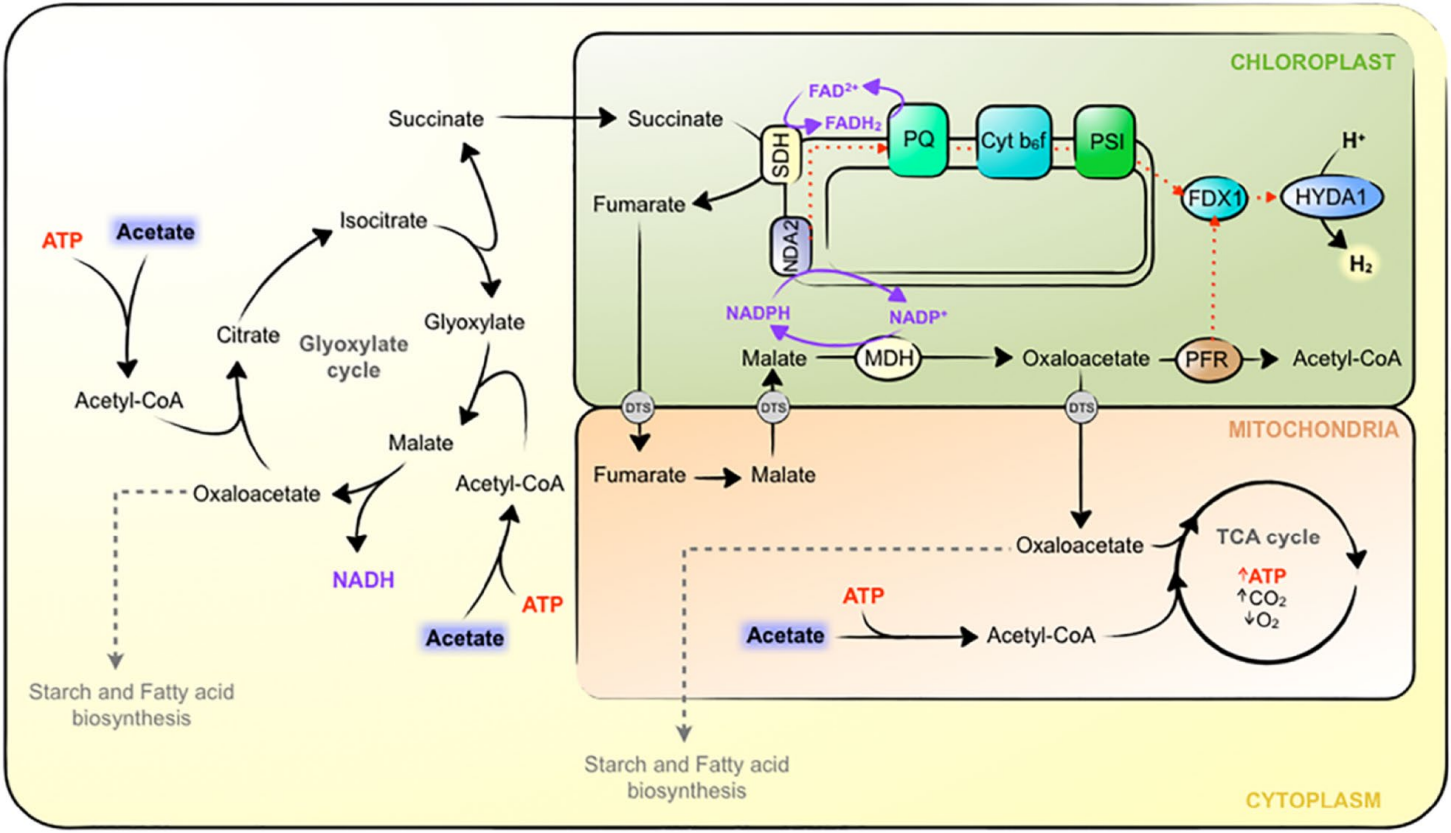
Tentative model for H_2_ production pathways in acetate-containing cultures under LL. *Black dashed arrows* multiple enzymatic steps. *Red dashed arrow* electrons flow. *DCT* dicarboxylate transport system, *Cytb6f* cytochrome b6f, *HYDA1* hydrogenase 1, *FDX1* ferredoxin 1, *MDH* malate dehydrogenase, *NDA2* NAD(P)H:plastoquinone oxidoreductase 2, *PFR* pyruvate:ferredoxin oxido reductase, *PSI* photosystem I, *PQ* plastoquinone, *SDH* succinate dehydrogenase, *TCA* tricarboxylic acid cycle

The H_2_ production observed in acetate-containing media is clearly enhanced in the light, although, under our experimental conditions, dark fermentation may contribute to about 10–20 % of the total H_2_ production detected at 12PAR. The stimulatory effect of light on H_2_ production necessarily requires an active photosynthetic electron flow that can feed electrons to HYDA1 via either the PSII-dependent or PSII-independent pathway. The inhibitory effect of DCMU on algal H_2_ photoproduction has frequently been used to differentiate between PSII-dependent and -independent pathways [7, 10, 48]. Previously, it was observed that the effect of DCMU on cells grown in nutrient-replete media was unclear. Some studies reveal no impact of DCMU on H_2_ photoproduction [49]. However, others observed substantial inhibition of both H_2_ production (90 %) and acetate uptake (66 %) [21, 24]. Here, we demonstrate that the addition of DCMU to nutrient-replete, acetate-containing cultures can have very different effects on both H_2_ production (e.g., at 12 PAR, inhibition of 72.4 % in non-aerated cultures vs 19.2 % in aerated cultures) and acetate uptake (e.g., at any light intensity, severe inhibition in non-aerated cultures vs slight inhibition in aerated cultures). The level of inhibition of H_2_ production by DCMU is subjected to the specific culture conditions, more specifically to O_2_ availability and acetate uptake. This may be problematic for determining the contribution of the PSII-dependent pathway on cells grown in media containing acetate. Experimental conditions (e.g. O_2_ availability, pre-culture conditions, purging processes, etc.) may affect the effect of DCMU addition on H_2_ production and in turn overestimate the contribution of the PSII-dependent pathway. This may partly explain some discrepancies found in the literature concerning the relative importance of this pathway. Nonetheless, we conclude that H_2_ production in LL mixotrophic cultures is mainly via PSII-independent pathway, since up to 80 % of the maximal H_2_ production can be obtained when PSII is inhibited (Figs. 2e, 4e). It has been proposed that acetate may enhance the PSII-independent H_2_ production pathway under nutrient-replete media by favoring the accumulation of starch during oxygenic conditions, which is degraded later during anaerobiosis [17, 24, 49, 50]. However, our data do not seem to support this possibility since we observe no correlation between starch accumulation/degradation patterns and H_2_ production under our experimental conditions (Figs. 3a, 5). Our data are in agreement with previous observations of starch-deficient mutants that can produce H_2_ under both S-replete and S-depleted conditions [10]. Alternatively, starch degradation may not be the source of reductive equivalents but rather acetate assimilation may provide the chloroplast with reductive equivalents for PSII-independent H_2_ photoproduction. The localization of two enzymes involved in acetate assimilation, succinate dehydrogenase (SDH) and malate dehydrogenase (MDH), in the chloroplast of Chlamydomonas, may provide this organelle with the reductive equivalents (FADH_2_ and NADPH, respectively) needed to feed the PQ pool [51, 52] (Fig. 6). Additionally, the oxaloacetate produced in the chloroplast from MDH can also potentially contribute to H_2_ production. Recently, it was shown that the pyruvate:ferredoxin oxidoreductase (PFR) enzyme of Chlamydomonas possesses an important affinity for oxaloacetate [53], which opens up the possibility that acetate, via the glyoxylate pathway, might also be coupled to fermentative H_2_ production (Fig. 6).

In any case, under our experimental conditions, the potential contribution of these pathways (either PSII-independent via SDH/MDH or fermentative via PFR) needs to be necessarily linked to light-dependent reactions since dark H_2_ production is clearly lower than in the light. For the SDH/MDH pathway, it is easy to explain the light dependence since electron flow from the PQ pool to HYDA1 would require an active PSI. At the same time, an active PSI would promote PQ re-oxidation and would allow the turnover of FAD^+^ and NADP^+^ needed to maintain SDH and MDH activities, respectively, which in turn would provide oxaloacetate to the chloroplast, leading to high PFR rates under anoxia. Still, the relative importance, if any, of these potential pathways for H_2_ production in nutrient-replete acetate-containing cultures, remains to be determined appropriately.

Enzyme localization and proteomics studies have helped in identifying the acetate assimilation and dissimilation pathways, which require the participation of enzymes localized in the cytosol, the mitochondria, and the chloroplast [51, 52, 54–56]. Based on these studies and on the physiological data we have provided in this study, we propose a tentative model that aims at explaining the physiology and metabolic pathways involved in H_2_ production in nutrient-replete, acetate-containing medium (Fig. 6). Acetate can be simultaneously routed to different pathways. Acetate can be dissimilated in the TCA cycle or assimilated by the glyoxylate cycle. The acetate entering the TCA cycle would provide energy to the cell and would contribute in maintaining low O_2_ levels. The glyoxylate cycle would provide succinate to the chloroplast for carbon skeletons. Chloroplastic SDH and MDH would participate in the conversion of chloroplast succinate into oxaloacetate and would provide reductive equivalents to the PQ pool. Chloroplastic oxaloacetate would either be redirected towards different biosynthetic pathways (e.g., lipids or starch biosynthesis) or used by chloroplast PFR if anoxic/hypoxic conditions are established. All these pathways occur simultaneously and their relative activities can be affected by O_2_ availability. Light intensity greatly affects O_2_ availability through PSII activity, and this O_2_ could be used by the TCA cycle to dissimilate acetate. If the light intensity is not too high, then the TCA cycle would be able to maintain the cells under hypoxic conditions. However, even though hypoxic conditions are established, O_2_ photoproduction rates would regulate the TCA cycle activity, acetate uptake rate, and cell growth. The higher these parameters, the lower would be the H_2_ production. Under LL, however, O_2_ availability is very low and so would be the TCA activity, acetate uptake rates, and ATP generation. Under this scenario, the relative importance of the glyoxylate cycle is enhanced, as well as chloroplastic SDH and MDH activities, which could provide reductive equivalents to the PQ pool. Also, due to low ATP levels, the use of oxaloacetate for biosynthetic pathways and cell growth is impaired and PFR activity is favored. All these processes would favor the H_2_ production under LL in acetate-containing medium. Under severe anoxic conditions, no acetate would be taken up, and H_2_ production would be low. Finally, in the dark, and independent of O_2_ availability, PSII-independent H_2_ production is not active, and the turnover of FADH_2_ and NADPH would limit oxaloacetate availability and its use by PFR.

## Conclusion

H_2_ production by Chlamydomonas cultures is still far for large-scale implementations due to the low yields obtained so far. Among other limitations, the H_2_ partial pressure is an important limiting factor, as shown in this work. Moreover, most of the studies about H_2_ production on Chlamydomonas rely in the use of nutrient starved cultures, which reduces the viability of large-scale productions. The approach presented here, although also has very low H_2_ production yields, opens up a new possibility to the straightforward attainment of both H_2_ and biomass in non-stressed cultures. This could have a great biotechnological interest if current H_2_ production yields are sufficiently improved. Another factor limiting H_2_ production in algae is the O_2_ sensitivity of the hydrogenases. However, an intriguing conclusion of this work is that low levels of O_2_ can actually benefit H_2_ production by the means of facilitating acetate uptake in mixotrophic cultures. However, the role of acetate during the H_2_ photoproduction in Chlamydomonas cultures remains uncertain. We have shown that H_2_ production in mixotrophic cultures is mostly associated with the PSII-independent pathway (80 %), indicating that a source of reductants is needed to feed the PQ pool. However, although acetate assimilation can favor starch accumulation, we clearly demonstrate that mobilization of the starch reserves does not provide with reductants for H_2_ production under our experimental conditions. We discuss potential metabolic pathways that may be involved in H_2_ production and linked to the dissimilation/assimilation of acetate, but certainly, more studies are needed to unravel the precise mechanisms that trigger H_2_ production in the presence of acetate in Chlamydomonas cultures. Understanding such mechanisms could help us improving H_2_ production efficiency through new physiological manipulations and genetic engineering.

## Methods

### Alga strain and growth conditions

Chlamydomonas (Chlamydomonas reinhardtii) strain 704 (cw15 arg7^+^ Nia1:Ars mt^+^) [57] was used in all the experiments. Cells were grown photomixotrophically under continuous light at 23 °C in liquid TAP medium. The initial acetic acid concentration was of 17.4 mM [58]. Algal cultures were grown to mid-log phase (10–12 × 10^6^ cells mL^−1^), harvested by centrifugation (3000*g* for 2 min) and resuspended in fresh TAP to a final concentration of 10 μg Chl mL^−1^ (~3 × 10^6^ cells mL^−1^). For experiments employing either TAP without S (TAP-S) or acetate free media (Minimal Media, MM) [58], cells were washed two times in the corresponding media before setting up the inductions. Cells (100 ml) were placed in a hermetically sealed vessel (140 ml) using screw caps equipped with silicon septa (GL45 PP 2 ports, GL14, Duran Group). The final headspace volume was 40 mL. The sealed vessels were placed in a growth chamber equipped with LED panels (AlgaeTron AG 230, Photon System Instruments) at 23 °C with continuous agitation (210 rpm). The following light intensities were assessed: 12, 22, 50, and 100 µE m^−2^ s^−1^. For dark inductions, the vessels were covered with aluminum foil. Where indicated, the following reagents were added to the cultures: the PSII inhibitor, DCMU (25 µM of 3-(3,4-dicholorophenyl)-1,1-dimethylurea in ethanol) (Sigma-Aldrich); 8.5 mM glacial acetic acid (Panreac); and 8.5 mM potassium acetate. Daily samples (1 ml) of the cultures were collected using a syringe; and they were used for starch, chlorophyll, acetate, and pH determinations.

### Analysis of the gases in the head spaces of the cultures

Samples (250 µL) from the headspaces of the cultures were collected using a 1 mL Hamilton’s SampleLock^TM^ syringe (# 81356) and manually injected into a Gas Chromatographer (GC) (Agilent 7820A, Agilent Technologies). H_2_, O_2_ and CO_2_ were detected using a Thermal Conductivity Detector (TCD), and argon was used as the carrier gas. H_2_, O_2_, and CO_2_ were separated at 40 °C using a two-capillary-column system (HP-molesieve 30 m x 0.53 mm and HP-PLOT/Q 30 m × 0.53 mm, Ref. 19095P-MS6/19095P-QO4, Agilent Technologies) coupled with a pneumatic valve. Alternatively, O_2_ and H_2_ were separated using a packed column (60/80 Molecular Sieve 5A, Ref. 13133-U, Supelco) at 70 °C.

### Chlorophyll, acetate, and starch measurements

Culture samples (1 mL) were taken directly from culture vessels at the indicated times, centrifuged for 2 min at 13,000 rpm, and the pellets were resuspended in methanol for chlorophyll (a + b) extraction [58]. Supernatants were stored at −20 °C separately for acetate determinations. The concentrations of chlorophylls a and b, extracted from cells in methanol, were estimated spectrophotometrically (DU 800, Beckman Coulter).

Acetic acid analysis was performed by HPLC (Agilent series 1200, Agilent Technologies) using an ion-exchange column (Agilent Hi-Plex H, 300 × 7.7 mm, 6 μm I.D.) and isocratic elution with 5 mM H_2_SO_4_ at 55 °C. Samples were thawed, centrifuged, and filtered prior to HPLC analysis. Twenty µl of cell culture supernatant was injected onto the HPLC system at a flow rate of 0.6 ml/min. Retention peaks were observed using a UV (205 nm) detector and recorded using Agilent ChemStation software. Quantifications were performed by comparisons with known amounts of standard

Starch extraction was performed using a version of the method of Klein and Betz [17]. One milliliter of culture was sampled, centrifuged at 13,000 rpm for 1 min, resuspended in 1 mL of methanol for chlorophyll extraction, and centrifuged again. The pellets were rinsed with 400 µL of distilled water and heated for 15 min at 120 °C for starch solubilization. Two hundred microliter of a mixture of α-amylase (A-9857, SIGMA) and amyloglucosidase (10115, SIGMA) containing 2.25 U/mL each, prepared in Na-acetate buffer (100 mM, pH 4.5), were added to the samples. The mixture reaction was incubated for 2 h at 55 °C. Finally, free glucose was measured using a BioSystems kit for glucose determination (11538, BioSystems)

## Additional files

**Additional file: Figure S1.** Chlorophyll evolution. A, non-aerated cultures; B, aerated cultures; C, aerated cultures supplemented with acetic acid; D, non-aerated cultures + DCMU; E, aerated cultures + DCMU; F, aerated cultures supplemented with acetic acid + DCMU. For conditions C and F acetic acid (8.5 mM) and DCMU supplementations were done on days 4 and 7. Represented data are average from at least three independent experiments.
**Additional file 2: Figure S2.**  Effect of daily O_2_ addition to hermetic cultures. H_2_ (A), O_2_ (B) levels where determined for the headspaces. Acetic acid (C) was determined in the media. Daily addition of 8.4 ml of pure O_2_ to the 40 ml headspaces (21 % of O_2_ into the headspace) was obtained through syringe injection across the septum vessels. Cultures were incubated under 12 PAR. For comparison purposes non-aerated 12 PAR cultures condition is also include in the graphic. Represented data are average from at least three independent experiments.
**Additional file3: Figure S3.** Determination of H_2_ in S-depleted cultures under 12 PAR. H_2_ measurements were done in non-aerated (A), aerated (B) and daily purged (C) cultures. The corresponding controls for S-replete conditions are also represented. For aerated and purged cultures H_2_ accumulative productions are plotted. Represented data are average from at least three independent experiments.

## Abbreviations

HL: high light (100 PAR)
HYDA1: hydrogenase 1
FDX1: ferredoxin 1
LL: low light (12-22 PAR)
MDH: Malate Dehydrogenase
ML: moderate light (50 PAR)
NDA2: NAD(P)H:plastoquinone oxidoreductase 2
PAR: photosynthetically active radiation (in µmol photons m^−2^s^−1^)
PFR: pyruvate:ferredoxin oxido Reductase
PQ: plastoquinone
SDH: succinate dehydrogenase
TAP: tris–acetate–Phosphate medium
TCA: tricarboxylic Acid cycle
