# Supplementary figures and images for: Low oxygen levels contribute to improve photohydrogen production in mixotrophic non-stressed Chlamydomonas cultures
Source: Biotechnol Biofuels. 2015 Sep 17;8:149. doi: 10.1186/s13068-015-0341-9 (PMC4573693; doi:10.1186/s13068-015-0341-9)

## Slide 1
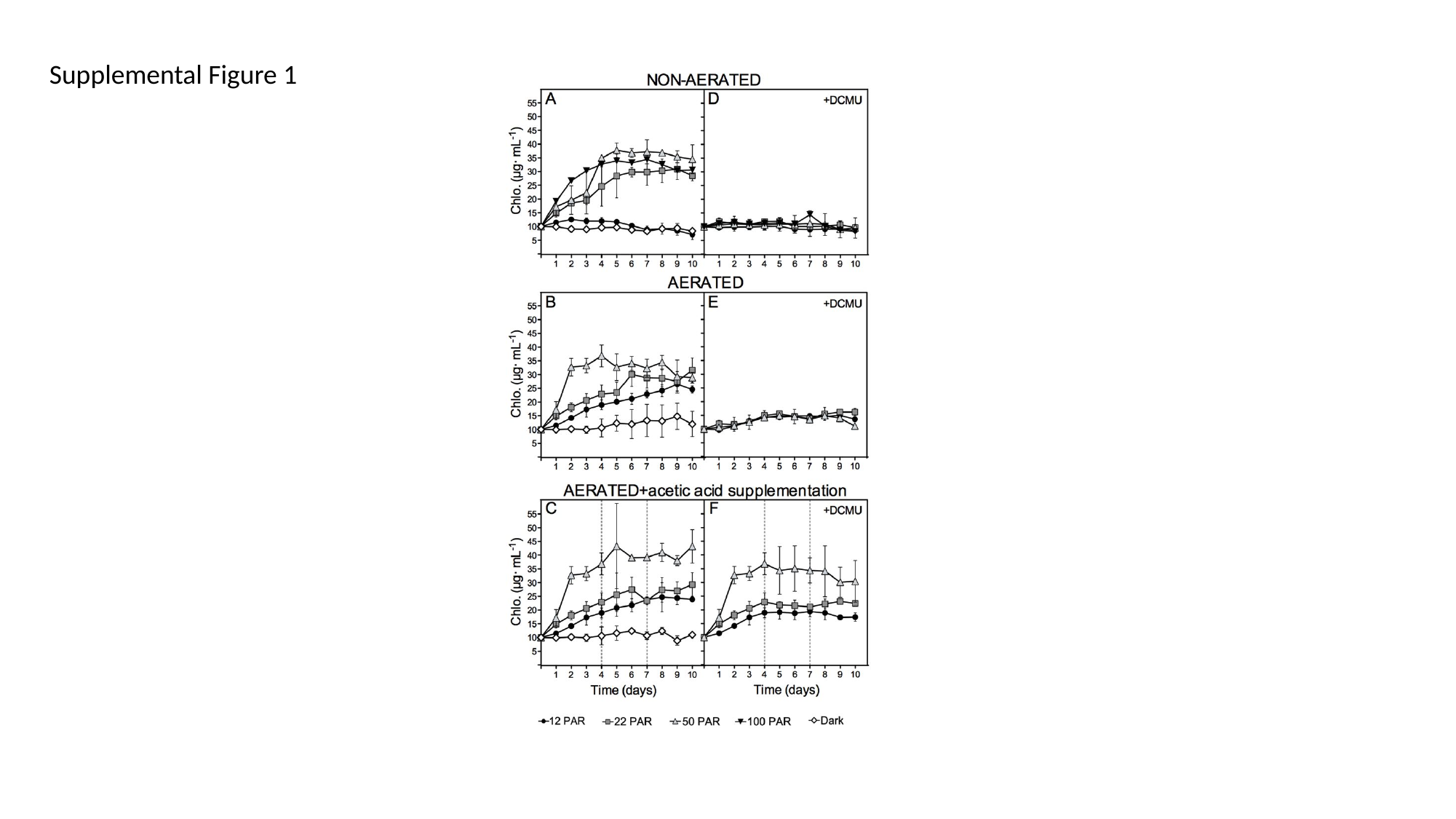

Supplemental Figure 1

Supplement: Supplementary file 1 — Additional file: Figure S1. Chlorophyll evolution. A, non-aerated cultures; B, aerated cultures; C, aerated cultures supplemented with acetic acid; D, non-aerated cultures + DCMU; E, aerated cultures + DCMU; F, aerated cultures supplemented with acetic acid + DCMU. For conditions C and F acetic acid (8.5 mM) and DCMU supplementations were done on days 4 and 7. Represented data are average from at least three independent experiments. [file 13068_2015_341_MOESM1_ESM.pptx]

## Slide 1
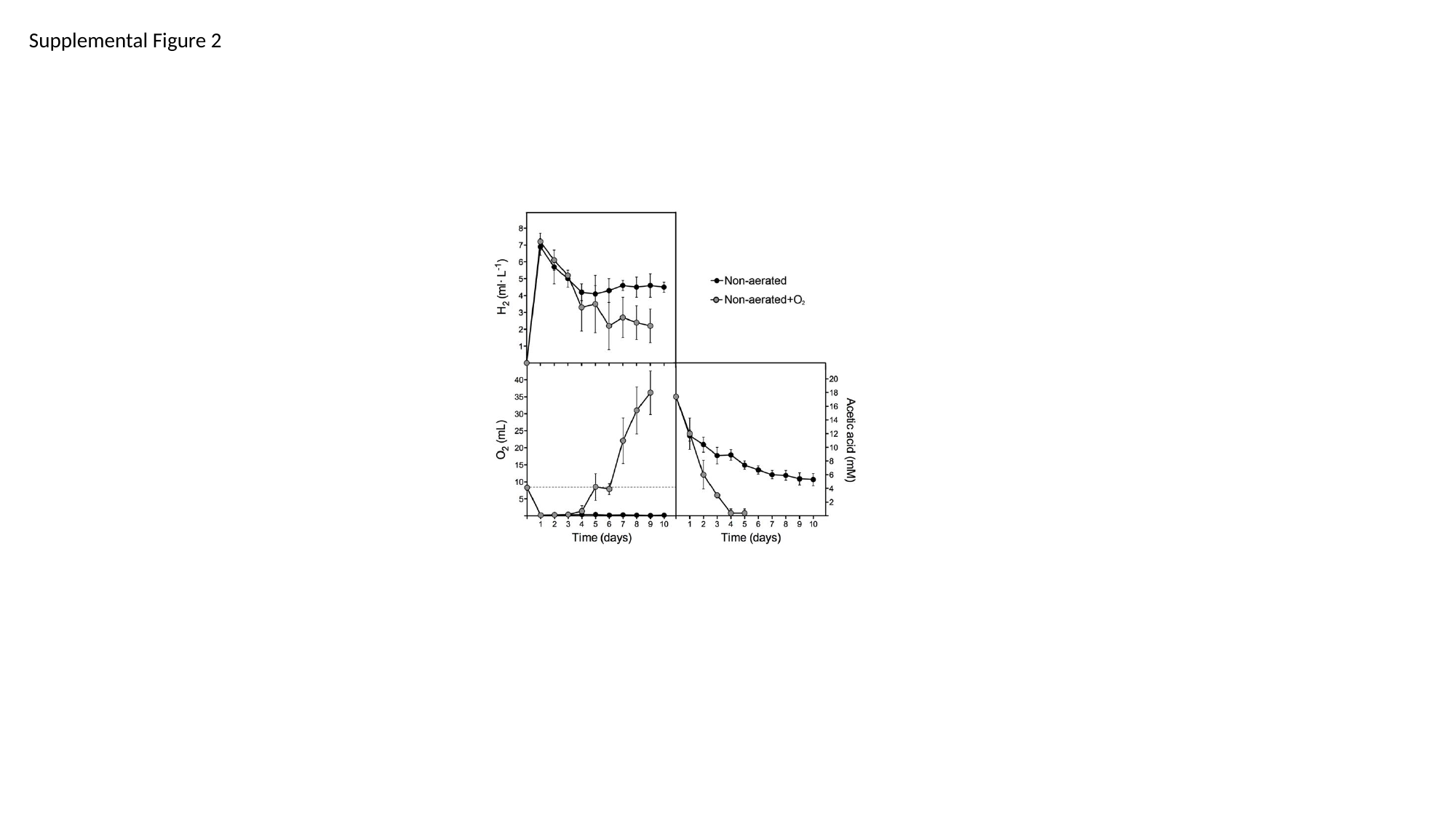

Supplemental Figure 2

Supplement: Supplementary file 2 — Additional file 2: Figure S2. Effect of daily O2 addition to hermetic cultures. H2 (A), O2 (B) levels where determined for the headspaces. Acetic acid (C) was determined in the media. Daily addition of 8.4 ml of pure O2 to the 40 ml headspaces (21 % of O2 into the headspace) was obtained through syringe injection across the septum vessels. Cultures were incubated under 12 PAR. For comparison purposes non-aerated 12 PAR cultures condition is also include in the graphic. Represented data are average from at least three independent experiments. [file 13068_2015_341_MOESM2_ESM.ppt]

## Slide 1
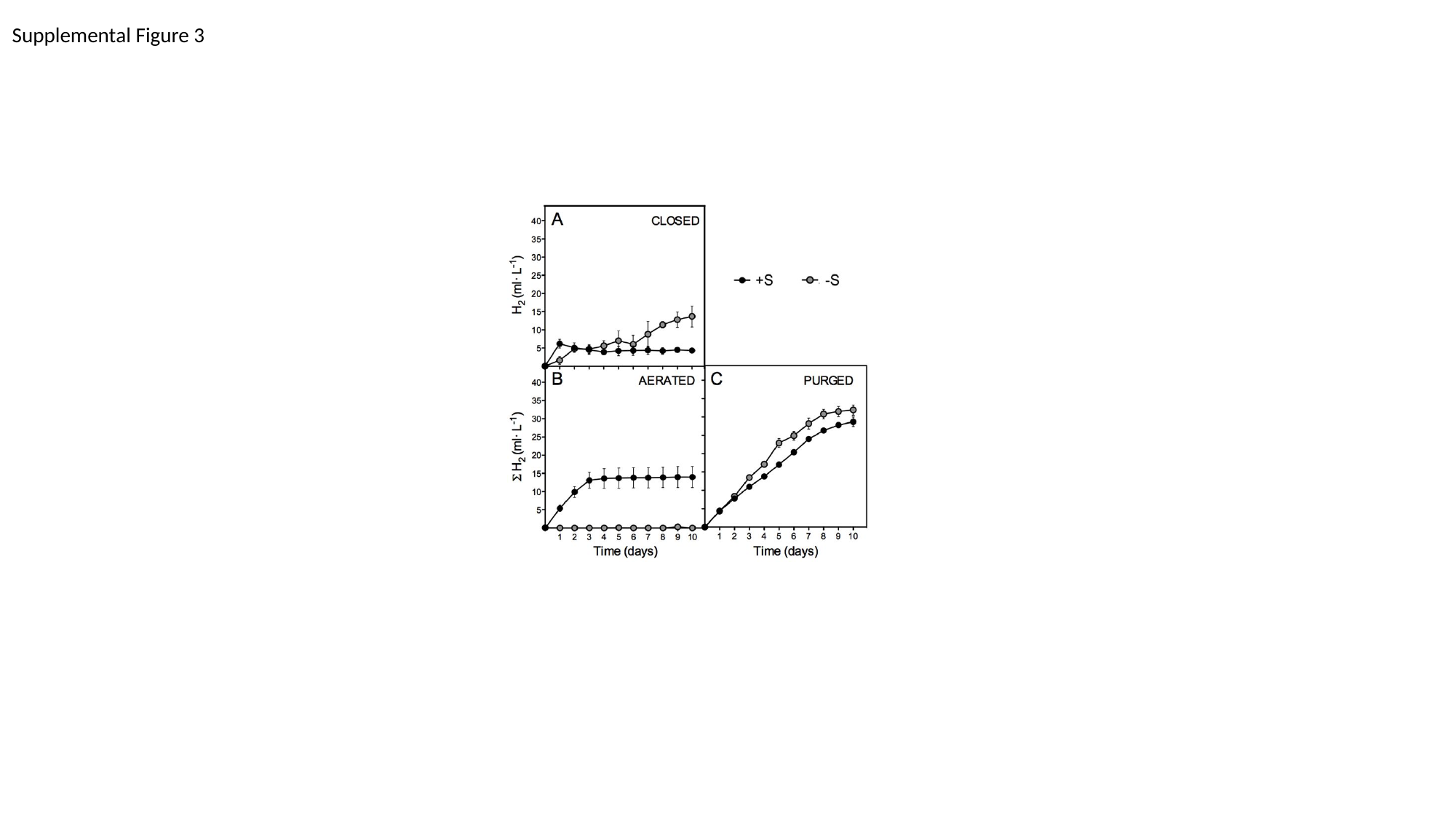

Supplemental Figure 3

Supplement: Supplementary file 3 — Additional file3: Figure S3. Determination of H2 in S-depleted cultures under 12 PAR. H2 measurements were done in non-aerated (A), aerated (B) and daily purged (C) cultures. The corresponding controls for S-replete conditions are also represented. For aerated and purged cultures H2 accumulative productions are plotted. Represented data are average from at least three independent experiments. [file 13068_2015_341_MOESM3_ESM.ppt]
